# Hippo pathway activation drives fibrogenic remodelling in influenza A virus-infected lung fibroblasts

**DOI:** 10.1183/23120541.01123-2025

**Published:** 2026-06-17

**Authors:** Yasmina Reisser, Elaine Winkler, Julia Hoffmann, Nilima Dinesh Kumar, Antje Häder, Susanne M. Lang, Bettina Löffler, Stefanie Deinhardt-Emmer

**Affiliations:** 1 Institute of Medical Microbiology, Jena University Hospital, Jena, Germany; 2 Else Kröner Graduate School for Medical Students “JSAM”, Jena University Hospital, Jena, Germany; 3 Clinic for Pneumology, Jena University Hospital, Jena, Germany; 4 These authors contributed equally

## Abstract

**Background:**

Pulmonary fibrosis is a progressive and often fatal interstitial lung disease with largely undefined aetiology. Alveolar macrophages and lung fibroblasts play key roles in maladaptive tissue remodelling; however, the cellular and molecular mechanisms underlying their coordinated fibrotic responses remain incompletely characterised.

**Methods:**

Following influenza A virus (IAV) infection, viral load, cytokine release and transcriptomic changes were analysed. To assess indirect effects on fibrosis, IMR-90 fibroblasts were treated with conditioned media from infected alveolar macrophage-like (AML) cells. Direct IAV infection of IMR-90 cells was also performed to evaluate changes in the Hippo signalling pathway and fibrotic marker expression using microarray and transcriptomic approaches.

**Results:**

For the first time, AML cells were successfully infected with IAV, validating this model for studying pathogen-driven exacerbation of fibrosis in ageing lungs. Transcriptomic and protein-level analyses revealed that IAV promotes fibrogenesis in fibroblasts through two distinct mechanisms: 1) indirectly, *via* pro-inflammatory and pro-fibrotic mediators released by infected macrophages; and 2) directly, through viral-induced modulation of the Hippo signalling pathway, resulting in upregulation of key fibrotic markers such as CTGF, fibronectin and collagen I.

**Conclusions:**

These findings provide novel mechanistic insights into macrophage–fibroblast crosstalk in the context of viral infection and fibrosis. They suggest that dysregulation of these interactions contributes to the susceptibility of the ageing lung to fibrotic remodelling following respiratory viral infections and may inform future therapeutic interventions targeting Hippo pathway signalling.

## Shareable abstract

**Macrophage-derived mediators and fibroblast Hippo signalling are critical drivers of virus-induced fibrosis, revealing actionable pathways for therapeutic intervention** https://bit.ly/4q6vOTa

## Graphical abstract

**Figure:**
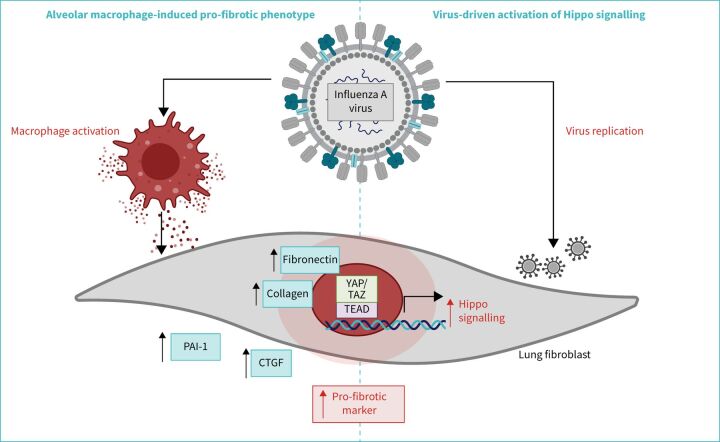
Influenza A virus (IAV) activates alveolar macrophages and directly engages Hippo signalling in lung fibroblasts, ultimately driving pulmonary fibrosis. YAP: Yes-associated protein; TAZ: transcriptional coactivator with PDZ-binding motif; TEAD: transcriptional enhanced associate domain; PAI: plasminogen activator inhibitor; CTGF: connective tissue growth factor.

## Introduction

Pulmonary fibrosis is a progressive and often fatal lung disease characterised by fibrotic remodelling and loss of respiratory function. Idiopathic pulmonary fibrosis (IPF), the most common and severe form of interstitial lung disease, is defined by progressive decline leading to respiratory failure [1, 2]. Its pathogenesis arises from repetitive epithelial injury and defective repair. Aberrant fibroblast activation drives myofibroblast differentiation, excessive extracellular matrix deposition and destruction of lung architecture [1, 3–7].

Overall, inflammation and fibrosis are the main pathological drivers of interstitial lung diseases (ILDs) [1]. Acute viral infections during disease exacerbations contribute to high morbidity and mortality, yet the impact of latent infections warrants further investigation [1, 8]. Influenza A virus (IAV), a major cause of acute respiratory infections [9], may exacerbate ILDs through persistent inflammation and dysregulated immune responses [10–13]. Moreover, accumulating evidence suggests that viral infections are not only drivers of disease exacerbation but may also contribute directly to the genesis of pulmonary fibrosis [14].

Alveolar macrophages (AMs) regulate fibrotic processes, with M1 macrophages promoting injury and M2 macrophages driving fibrosis further and promotes fibrosis progression [15–17]. Additionally, macrophages interact with fibroblasts and contribute to their activation of pro-fibrotic factors [18, 19]. The Hippo signalling pathway, *via* Yes-associated protein (YAP) and transcriptional co-activator with PDZ-binding motif (TAZ), regulates fibroblast activation and ECM production [20] and contributes to fibrosis in multiple organs [21–23]. Dysregulation of this pathway has been implicated in fibrosis, specifically by promoting fibroblast proliferation, myofibroblast differentiation and ECM-protein deposition [24, 25]. Additionally, IAV has been identified as an activator of YAP and TAZ *via* their interaction with the viral NS1 protein in lung epithelial cells as well as murine AT1 and AT2 cells [23, 26], suggesting a link between viral infection and fibrosis.

In this context, our study focuses on human lung fibroblasts, which we previously identified as a relevant replication site contributing to IAV infection [27]. Therefore, we aim to investigate how IAV-induced alterations in the Hippo signalling pathway drives fibrotic remodelling in lung fibroblasts, shedding light on the mechanisms by which respiratory viral infections may exacerbate pulmonary fibrosis.

## Materials and methods

### Patients

10 patients undergoing bronchoscopy at Jena University Hospital (October 2023–September 2024) were screened, and gave informed consent for use of remaining bronchoalveolar lavage (BAL) material (six fibrotic ILD, four sex-matched nonfibrotic controls (table 1), confirmed by expert interstitial lung board). The study was approved by the ethics committee of Friedrich Schiller University Jena (No: 2024-3391). BAL was performed *via* flexible bronchoscopy under sedation, usually targeting the middle lobe. About 100 mL body-warmed saline was instilled, with 50–90% fluid retrieved; residual material was used for research.

**TABLE 1:** Overview of patient characteristics and clinical parameters

	Nonfibrotic				Fibrotic					
	Patient 1	Patient 2	Patient 3	Patient 4	Patient 1	Patient 2	Patient 3	Patient 4	Patient 5	Patient 6
**Age years**	54	64	76	73	68	60	65	76	64	61
**Sex**	Male	Male	Male	Male	Male	Male	Male	Male	Male	Male
**Weight kg**	78	140	80	60	83	129	91	98	140	106
**BMI kg·m^−2^**	26	44	26	23	25	39	24	31	39	35
**Smoking status**	Former	Former	Former	Never	Former	Never	Former	Former	Former	Former
**Pack-years (smoking exposure)**	10	40	15	0	20	0	40	15	30	5
**Immunosuppressive drugs**	None	None	None	None	None	None	None	None	None	None
**Lung disease diagnosis**	ILA	Emphysema	Emphysema	EGPA Asthma	ILD (not classifiable) diaphragm protrusion	IPF	NSIP	ILD (not classifiable) COPD	ILD (not classifiable) OSAS	ILD (not classifiable) OSAS
**Radiological pattern**					Mixed	UIP	NSIP	Possible UIP	Possible UIP	Mixed
**White blood cell count Gpt·L^−1^**	10.40	6.30	6.20	5.30	5.90	13.70	8.20	7.50	8.90	4.60
**Interleukin-2 level U·mL^−1^**	371	488	NA	NA	419	407	NA	NA	NA	NA
***D*_LCO_** **mmol·min^−1^·kPa^−1^**	9.29	6.95	6.24	5.49	6.57	3.06	4.17	3.90	8.26	9.66
***D*_LCO_ % predicted value**	97	73	76	81	69	30	38	45	80	104

ILA: interstitial lung abnormalities; EGPA: eosinophilic granulomatosis with polyangiitis; ILD: interstitial lung disease; IPF: idiopathic pulmonary fibrosis; NSIP: nonspecific interstitial pneumonia; OSAS: obstructive sleep apnoea syndrome; UIP: usual interstitial pneumonia; *D*_LCO_: diffusing capacity of the lungs for carbon monoxide.

### Cell culture

IMR-90 human lung fibroblasts (Coriell Institute, USA) were cultured in DMEM (Sigma Aldrich) with 10% FBS (PAN Biotech) and 1% penicillin/streptomycin (P/S, Lonza). Madin-Darby canine kidney (MDCK) cells were maintained in EMEM (Sigma Aldrich) with 10% FBS and 1% P/S. Patient-derived AMs were isolated from BAL fluid, adapted from a previously published protocol [28].

Hippo Pathway TEAD Luciferase Reporter MCF7 cells (Biomol) were cultured in EMEM with 10% FBS, 1% NEAA, 1 mM sodium pyruvate, 1% P/S, 400 µg·mL^−1^ Geneticin and 10 µg·mL^−1^ insulin. All cells were grown at 37°C and 5% CO_2_. For fibroblast-to-myofibroblast transformation, IMR-90 cells were treated on day 3 with 1.25 ng·mL^−1^ transforming growth factor-β1 (TGF-β1); quiescent controls were generated by serum starvation [29].

Peripheral blood mononuclear cells (PBMCs) were isolated from buffy coats by density centrifugation on Histopaque-1077 (Sigma Aldrich) subsequent washing steps, as detailed in the supplementary material. Monocytes were purified from PBMCs using CD14^+^ magnetic bead selection (Miltenyi Biotec) following the manufacturer's instructions.

### Alveolar macrophage-like cells

Monocyte-derived macrophages (MDMs) were generated by culturing purified monocytes in RPMI 1640 with 10% human serum (Sigma-Aldrich), 1% P/S and 10 ng·mL^−1^ granulocyte–macrophage colony-stimulating factor (GM-CSF) (Biolegend) for 6 days. Alveolar macrophage-like (AML) cells were differentiated as described [30] in RPMI 1640 with 10% human serum, 1% P/S and a cytokine/surfactant cocktail. MDMs and AMLs were cultured at 2×10^6^ cells·mL^−1^ in anti-adherence suspension plates (Greiner Bio-One) at 37°C, 5% CO_2_, with media changes every 3 days. After 6 days, cells were seeded at 0.5×10^6^ cells·mL^−1^ with differentiation media for follow-up experiments. Detailed experimental procedures are available in the supplementary material.

### Viral propagation and plaque assay

Infection experiments were performed using Influenza A virus/H1N1/Puerto Rico/8/1934 (IAV/PR8). IAV was propagated in MDCK cells in EMEM containing 10% FBS and 1% P/S. When cytopathic effects became visible, supernatants were collected, centrifuged at 4°C, and stored in aliquots at −80°C. Virus titre was determined by plaque assay on MDCK cells to quantify plaque-forming units, as described previously [31].

### Infection and conditioned media experiments

IAV infections were performed at a multiplicity of infection (MOI) of 1 for AM, and MOIs of 0.1 and 1 for IMR-90 and TEAD Reporter-MCF7 cells, as indicated. Cells were incubated with virus diluted in infection DPBS for 30 min and harvested at the indicated time points.

The cells were washed once with RPMI 1640 and then incubated with the virus in RPMI without supplements for 30 min at 37°C in 5% CO_2_. Following the virus incubation, cells were cultured in RPMI 1640 containing 10% human serum and the differentiation cocktail as described above for 8 and 24 h.

Conditioned media (CM) was collected from differentiated AML cells either uninfected (after 2 additional days of culture) or following IAV infection for 8 or 24 h. Supernatants from infected AMLs were UV-inactivated for 30 min prior to use. IMR-90 cells were treated with the respective media for 8, 24 or 48 h.

### TEAD activity measurement

Hippo Pathway TEAD Reporter MCF7 cells were treated with ONE-Step Luciferase Assay System (BPS Bioscience) according to the manufacturer's protocol. Luminescence was measured using the Infinite M-Plex Tecan reader (Tecan). Okadaic acid (BPS Bioscience) was used as a positive control.

### TEAD inhibition

IMR-90 cells were seeded 1 day prior to treatment and subsequently incubated with 250 nM or 500 nM K-975 (TEAD inhibitor; Selleckchem) for 48 h. Control cells were treated with equivalent concentrations of DMSO. Cells were then infected as described above at a MOI of 0.1 for 24 h with K-975 in the infection medium.

### RNA extraction, cDNA synthesis and real-time quantitative reverse transcription PCR

Total RNA was isolated using the RNeasy Mini Kit (Qiagen) and reverse transcribed with the High-Capacity cDNA Kit (Thermo Fisher Scientific). Real-time quantitative reverse transcription PCR (qRT-PCR) was performed with Maxima SYBR Green Master Mix (Thermo Fisher Scientific) on a Rotor-Gene Q cycler (Qiagen). Primer sequences are listed in supplementary table S1.

Viral RNA from AML cell supernatants and lysates were extracted with the QIAamp Viral RNA Mini Kit (Qiagen). IAV qRT-PCR was conducted using the RIDA GENE flu kit (R-Biopharm) on a Rotor-Gene Q according to the manufacturer's protocol.

### mRNA sequencing

RNA of patient-derived AM (per group: n=3) and AML cells (per group: n=4) was isolated, and concentration was determined. Novogene Co., LTD (Beijing) carried out library construction and mRNA sequencing. Sequencing was performed employing the Illumina platform NovaSeq 6000 S4 flowcell v1.0 with a PE150 strategy based on sequencing by synthesis (SBS). The bioinformatic analysis is outlined in the supplementary material.

### Protein detection

Cells were lysed in RIPA buffer containing Halt Protease and Phosphatase Inhibitor Cocktail (Thermo Fisher Scientific). Protein concentrations were determined using the Micro BCA Protein Assay Kit (Thermo Fisher Scientific). Extracted proteins were separated by SDS-PAGE and transferred onto polyvinylidene fluoride membranes (Thermo Fisher Scientific). The supplementary material contains primary and secondary antibodies, along with an expanded workflow. After incubation with HRP-conjugated secondary antibodies, membranes were visualised and quantified using the iBright CL750 Imaging System (Thermo Fisher Scientific). CTGF and PAI 1 ELISAs (SimpleStep ELISA Kit, abcam) were performed using cell culture supernatants according to the manufacturer's protocol.

### Immunofluorescence

AML cells were stained with primary (IAV Nucleoprotein) and appropriate secondary antibodies as outlined in the supplementary material. Visualisation was carried out using the All-in-One Fluorescence Microscope BZ-X810 (Keyence).

### Flow cytometry

MDM and AML cells were detached and stained with BD Fixable Viability Stain 780 (BD Biosciences) to assess viability. Cells were then blocked and incubated with antibodies targeting MARCO, CD36 and CD11b (supplementary table S1).

Secreted cytokines were quantified using LEGENDplex panels (Anti-Virus Response Panel 1 V02, Essential Immune Response Panel and Immune Checkpoint Panel 1; BioLegend). Samples were analysed on a BD Symphony A1 Cytometer (BD Biosciences), and data processed with Qognit v2023-02-15 or FlowJo v10.8.1.

### Hippo pathway screening array assay

IMR-90 cells were lysed in RIPA buffer supplemented with Halt Protease and Phosphatase Inhibitor Cocktail. A semi-quantitative analysis of 118 Hippo signalling-associated proteins was conducted by Creative Hippo Pathway Screening Array Assay (Shirley, New York, USA) using a pathway-specific antibody array. Further methodological details are provided in the supplementary material.

### Statistical analysis and design

Statistical analyses were conducted in GraphPad Prism 10. Nonparametric tests (Mann–Whitney U-test, Kruskal–Wallis and Dunn's multiple comparison) or parametric unpaired t-tests were applied as indicated in the figure legends. Where applicable, outliers were identified and removed using the ROUT method. Figures were created using Biorender.com.

## Results

### AMs from fibrotic patients exhibit a heightened inflammatory phenotype upon infection

**FIGURE 1:**
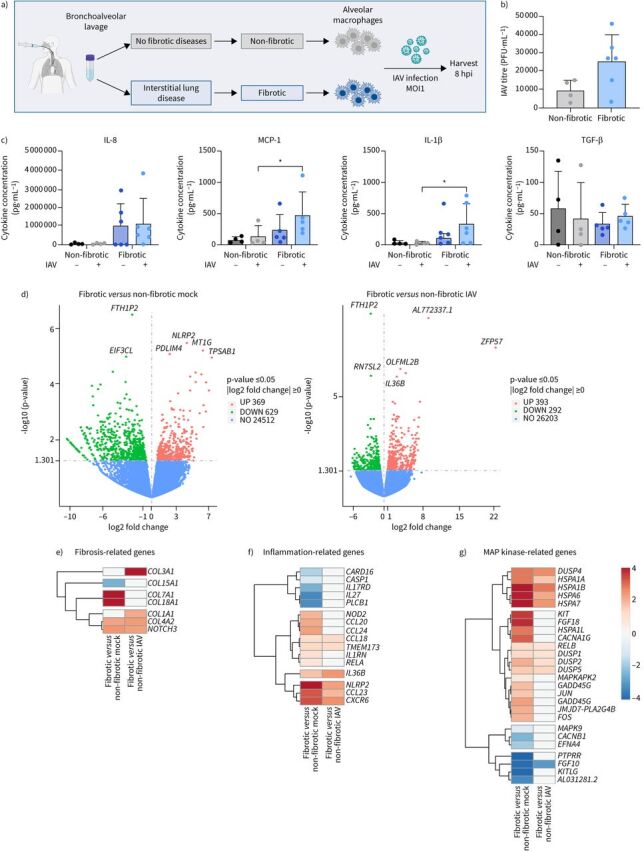
Influenza A virus (IAV) infection of alveolar macrophage (AM) from fibrotic patients results in elevated viral load and inflammatory response. a) Schematic overview of the study design. Patients who received a bronchoalveolar lavage (BAL) were grouped in fibrotic (n=6) and nonfibrotic (n=4) groups based on clinical data. AM were subsequently isolated from the BAL and infected with IAV with a multiplicity of infection (MOI) of 1 for 8 h. b) Viral titre in the supernatant of AM from fibrotic (n=6) and nonfibrotic (n=4) patients at 8 hpi, determined by plaque assay. IAV titers were normalised to cell count and expressed as PFU·mL^−1^, adjusted to 200 000 cells. c) Cytokine levels in the supernatant of mock- and IAV-infected AMs from fibrotic (n=6; unless outliers were removed) and nonfibrotic patients (n=4; unless outliers were removed) were measured. Values were normalised to cell count and expressed as pg·mL^−1^, adjusted to 200 000 cells. d) Volcano plots displaying differentially expressed genes (DEGs), with upregulated genes shown in red and downregulated genes in green. For each comparison, the six most significant DEGs are highlighted. Significance was defined as p≤0.05. Three biological replicates of patient-derived AMs were sequenced per group. e–g) Heatmaps depicting the log2 fold changes of DEGs comparing fibrotic and nonfibrotic samples under mock- and IAV-infected conditions. Genes associated with fibrosis (e), inflammation (f) and the MAP kinase pathway (g) are shown. Upregulated genes are displayed as red, while downregulated genes are displayed as blue. Log2 fold change of genes, which were not differentially expressed, was set to 0 and are displayed as white. Each data point represents an individual biological replicate. Data are shown as mean±sd. Statistical significance was assessed using the Mann–Whitney U-test (b) and Kruskal–Wallis test (c) (*p≤0.05). For c, outliers were identified and removed using the ROUT method. PFU: plaque-forming unit; hpi: hours post-infection; IL: interleukin; TGF-β: transforming growth factor-β.

We investigated AMs from six patients with fibrotic ILD and four patients with nonfibrotic lung diseases (sex-matched, table 1). To assess their response to viral infection, AMs were infected with IAV (H1N1/Puerto Rico/8/1934). Viral replication tended to be higher in AMs from fibrotic patients, although this difference did not reach statistical significance (figure 1b). Upon infection, fibrotic AMs released significantly more MCP-1 and interleukin (IL)-1β, cytokines known to promote fibrotic remodelling and fibroblast activation (figure 1c, supplementary figure S1A) [32].

To explore underlying molecular differences, we performed mRNA sequencing, which revealed numerous differentially expressed genes (DEGs) between fibrotic and nonfibrotic AMs in both mock- and IAV-infected conditions. *FTH1P2* was consistently regulated across both comparisons, while additional DEGs were unique to each condition (figure 1d). Analysis of specific gene subsets showed upregulation of fibrotic genes such as *COL4A2*, *COL7A1* and *COL1A1*, as well as pro-fibrotic chemokines including *CCL24* and *CCL18*, and inflammasome-related factors such as *NLRP2* in fibrotic AMs (figure 1e–f). Notably, the anti-fibrotic cytokine-gene *IL27* was downregulated, and MAPK pathway genes were elevated even in the absence of infection, indicating a heightened baseline activation in fibrotic AMs (figure 1g).

Overall, these findings suggest that AMs from patients with lung fibrosis exhibit an intrinsically pro-fibrotic phenotype and a heightened pro-inflammatory response to infection, which may contribute to increased viral susceptibility and sustained inflammation in the fibrotic lung.

### Influenza A virus infection in AML cells indicates strong inflammation

**FIGURE 2:**
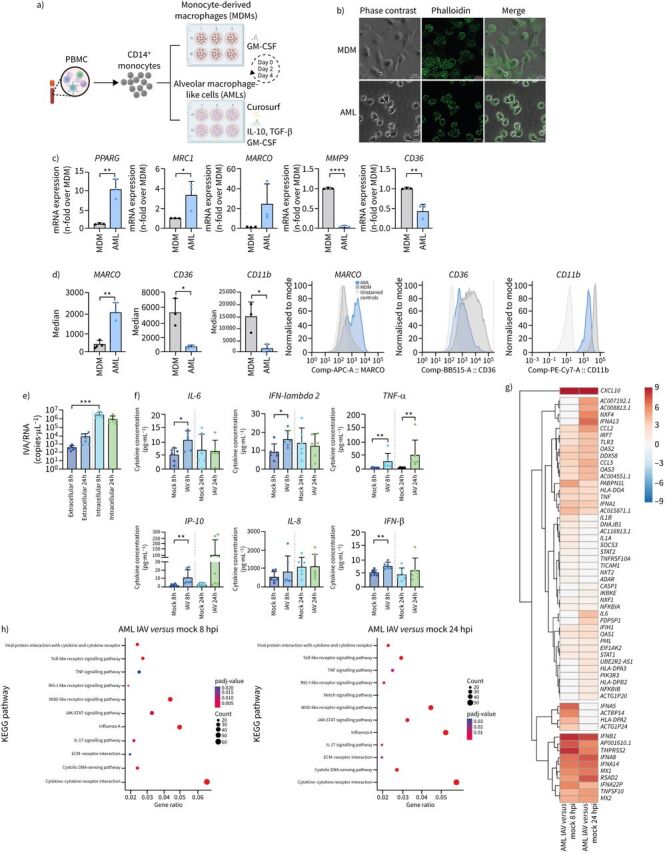
Characterisation of alveolar macrophage-like (AML) phenotype and immune response to Influenza A virus (IAV) infection. a) Schematic representation of differentiation of AML cells. Peripheral blood mononuclear cells (PBMCs) were isolated, and CD14^+^ monocytes were purified. Cells were cultured with granulocyte–macrophage colony-stimulating factor (GM-CSF) to generate monocyte-derived macrophages (MDMs). For AML cell differentiation, cells were cultured with a complete differentiation cocktail containing GM-CSF, transforming growth factor-β (TGF-β), interleukin (IL)-10, and Curosurf. Cytokines were added on days 0, 2, and 4, and cells were differentiated for a total of 6 days. b) Phase-contrast images show the morphology of AML cells compared to MDMs, along with phalloidin staining (green). Scale bar: 20 µM. c) Real-time quantitative reverse transcription PCR (qRT-PCR) analysis of *PPARG*, *MRC1*, *MARCO*, *MMP9* and *CD36*. Data are normalised to actin, and relative mRNA expression is plotted (n=3). d) Flow cytometry analysis of MARCO, CD36 and CD11b in AML cells compared to MDMs. Each dot represents an individual donor (n=3). Differentiated AML cells were either mock treated or infected with IAV (MOI1) for 8 and 24 h, and e) quantification of IAV RNA was performed in both extracellular supernatants and intracellular cell lysates (n=6). f) Cytokine levels in infected AML cells were measured using a multiplexed cytokine assay (n=6). g) Heatmap depicting log2 fold changes of differentially expressed genes (DEGs) related to IAV infection in the indicated comparisons. Upregulated genes are shown in red, downregulated genes in blue, and nondifferentially expressed genes in white (log2fold change=0). RNA sequencing was performed on four biological replicates of AML cells per group. h) Upregulated Kyoto Encyclopedia of Genes and Genomes (KEGG) pathways for the indicated comparisons. Each group included six biological replicates for sequencing. Data are expressed as mean±sd, and statistical significance was determined using an unpaired t-test (c, d), Mann–Whitney U-test (f) and Kruskal–Wallis and Dunn's multiple comparison test (e). *: p≤0.05, **: p≤0.01, ***: p≤0.001, ****: p≤0.0001. CD14^+^: cluster of differentiation 14 positive; MOI: multiplicity of infection; TNF-α: tumour necrosis factor-α; IFN: interferon.

To investigate the inflammatory response of AMs during IAV infection in a human-specific model, we utilised AML cells (figure 2a) [30, 33]. For this, PBMCs from healthy donors were differentiated under two conditions: with GM-CSF, TGF-β, IL-10 and curosurf to generate AML cells, or with GM-CSF alone to produce MDMs. AML cells maintained a rounded morphology and expressed higher PPAR-γ, MRC1 and MARCO, with lower MMP9 and CD36 levels compared to MDMs (figure 2b–d), confirming successful differentiation.

Cytokine profiling revealed that MDMs secreted more tumour necrosis factor and IL-6, whereas AML cells produced higher IL-10 and lower IFN-α2, MCP-1 and IL-23. Upon IAV infection, AML cells showed increased intracellular viral RNA and positive nucleoprotein staining, indicating robust infection (figure 2e, supplementary figure S2C). Infection triggered substantial upregulation of pro-inflammatory cytokines and chemokines, including *TNF*, *IL6*, *CXCL10*, *IL1B*, *CCL2* and *CCL5* (figure 2f,g, supplementary figure S2E). Transcriptomic analysis revealed modulation of type I interferons and ISGs (*MX1*, *OAS*, *RSAD2*), along with activation of immune pathways such as *TLR*, NOD-like receptor and JAK-STAT signalling (figure 2g–h). Notably, TGF-β secretion remained unchanged (supplementary figure S2F).

These results validate the AML model as a reliable tool to study alveolar macrophage responses to IAV, capturing both antiviral defense and inflammatory signalling.

### Inflammatory response of AML cells to IAV infection leads to upregulation of fibrosis markers in lung fibroblasts

Following validation of the AML cell model, we assessed the effect of AML-derived factors on pro-fibrotic responses in IMR-90 fibroblasts. Cells were treated with DMEM (control, basal IMR-90 medium), AML media or AML-conditioned media (CM) collected after 2 days of culture (supplementary figure S3A). Cytotoxicity remained low (LDH <15%), indicating cell viability (supplementary figure S3B). AML media and CM, containing TGF-β, significantly increased Collagen 1 and Fibronectin levels, suggesting that secreted mediators contribute to baseline fibrotic activation (supplementary figure S3C–D).

**FIGURE 3:**
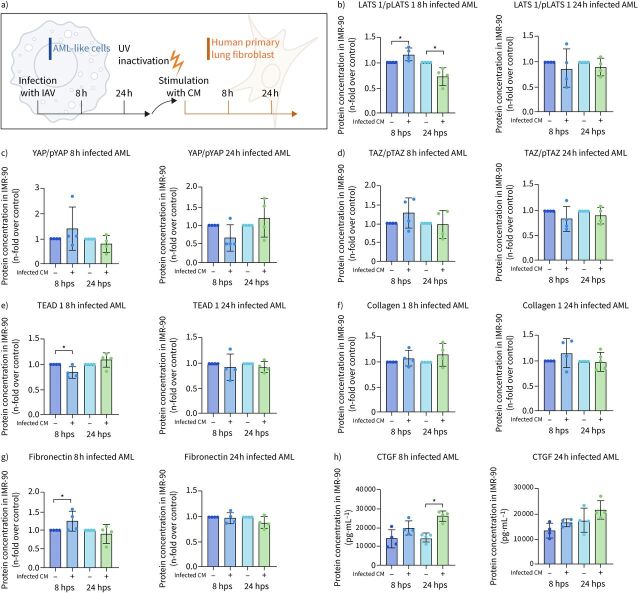
Inflammatory reaction of alveolar macrophage-like (AML) cells after Influenza A virus (IAV) infection leads to upregulation of fibrosis markers in lung fibroblast treated with conditioned media (CM) of AML cells. a) Schematic illustration of CM experiment. AML cells were infected with IAV with a multiplicity of infection (MOI) of 1 for 8 and 24 h. Collected and UV-inactivated supernatants were used to stimulate IMR-90 cells for 8 and 24 h. b–d) Ratio of phosphorylated to-unphosphorylated Hippo pathway proteins in IMR-90 cells treated with CM (n=4). Protein levels were quantified using Western blot analysis and expressed as fold change relative to the mock-infected control. e) TEAD 1 protein levels in IMR-90 cells treated with CM (n=4). Protein levels were quantified using Western blot analysis and expressed as fold change relative to the mock-infected control. f–g) Collagen 1 and Fibronectin protein levels in CM-treated IMR-90 cells (n=4). Protein levels were quantified using Western blot analysis and expressed as fold change relative to the mock-infected control. h) CTGF protein levels in CM-treated IMR-90 cells were measured using ELISA (n=4). Data are expressed as mean±sd, and statistical significance was calculated using Mann–Whitney U-test (b–h). *: p≤0.05. TEAD 1: TEA domain family member 1; CTGF: connective tissue growth factor.

To examine the effect of infection, IMR-90 cells were stimulated with CM from IAV-infected AML cells. Hippo signalling pathway proteins were analysed as potential regulators of virus-induced fibrosis. LATS1 was transiently activated at 8 h post-stimulation with infected CM, followed by downregulation at 24 h. No consistent changes were observed in YAP/TAZ, while TEAD1 was transiently downregulated at 8 h (figure 3b–e, supplementary figure S3E–H).

Fibrotic marker analysis revealed a trend towards higher Collagen 1 and significantly increased Fibronectin levels at early time points after treatment with infected CM (figure 3f–g), accompanied by elevated CTGF levels (figure 3h). Overall, CM from infected AML cells promotes early fibrotic activation in IMR-90 cells, without a clear involvement of Hippo signalling.

### IAV infection in lung fibroblast leads to an activation of YAP/TAZ

**FIGURE 4:**
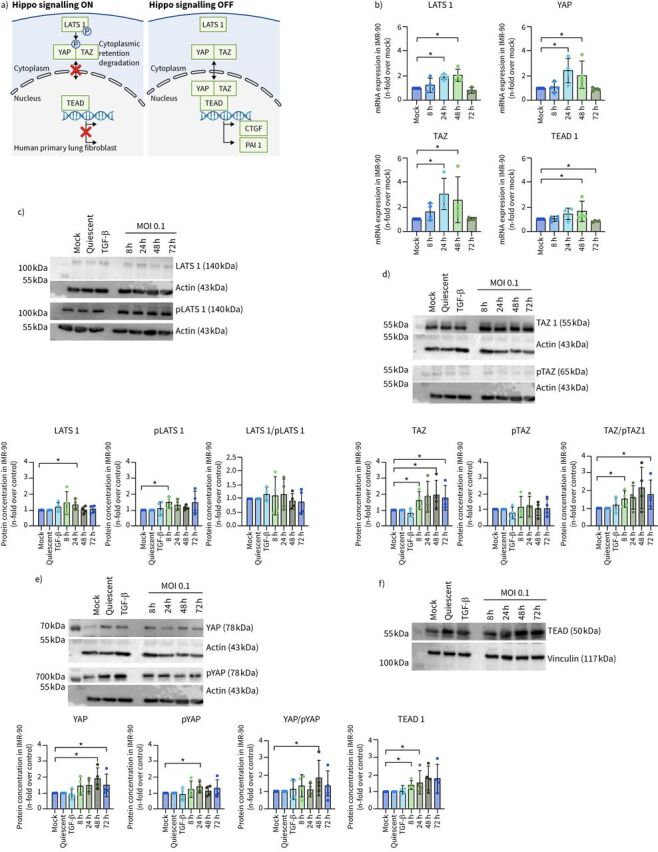
Influenza A virus (IAV) infection in lung fibroblast leads to an activation of YAP/TAZ and TEAD 1. a) Schematic illustration of Hippo signalling pathway. b) IMR-90 cells were infected with IAV with an multiplicity of infection (MOI) of 0.1. Hippo signalling factor expression was detected in qPCR (n=4). c–e) Protein levels of Hippo signalling proteins in infected IMR-90 cells were quantified by Western blotting and expressed as fold change relative to the respective control (n=4). The ratios of phosphorylated to unphosphorylated proteins are additionally provided. Representative blots are included. f) Protein levels of TEAD 1 in infected IMR-90 cells were quantified by Western blotting and expressed as fold change relative to the respective control (n=4). Data are expressed as mean±sd, and statistical significance was calculated using Mann–Whitney U-test (b–f). *p≤0.05. YAP: Yes-associated protein; TAZ: transcriptional coactivator with PDZ-binding motif; LATS 1: large tumour suppressor kinase 1; TEAD 1: TEA domain family member 1; CTGF: connective tissue growth factor.

In order to investigate the direct effect of IAV on fibrosis development and Hippo signalling in human lung fibroblast, IMR-90 cells were infected with IAV (supplementary figure S4A). Overall, the Hippo signalling pathway is regulated by phosphorylation and when inactivated, dephosphorylated YAP/TAZ translocate to the nucleus, interact with TEAD 1 and induce target gene transcription, including *CTGF* and *PAI1* (figure 4a). To analyse the effect on the Hippo signalling, the expression of *YAP*, *WWTR1 (encoding TAZ)*, *LATS1* and *TEAD1* was measured (figure 4b). Interestingly, *YAP*, *WWTR1* and *LATS1* were significantly upregulated after 24 and 48 hpi. TEAD 1 showed significant upregulation after 48 hpi, followed by a downregulation after 72 hpi; thus, indicating a time-dependent inactivation of the pathway by IAV. Western blot analysis was conducted to assess phosphorylation status. No significant changes were detected in the active/inactive ratio of LATS 1 (figure 4c). In contrast, the unphosphorylated (active) form of TAZ was significantly upregulated at multiple time points, with the active-to-inactive ratio showing a clear shift towards the activated state (figure 4d). The unphosphorylated as well as the phosphorylated form of YAP was significantly increased. However, the active-to-inactive ratio again revealed a clear shift towards the activated form at 48 hpi (figure 4e). Additionally, TEAD 1 levels were significantly elevated in infected IMR-90 cells after 8 and 24 hpi (figure 4f).

Collectively, these findings provide strong evidence that IAV infection in IMR-90 cells activates YAP/TAZ and their downstream target.

### IAV infection in lung fibroblast leads to higher levels of fibrotic markers indicating a fibrotic phenotype

**FIGURE 5:**
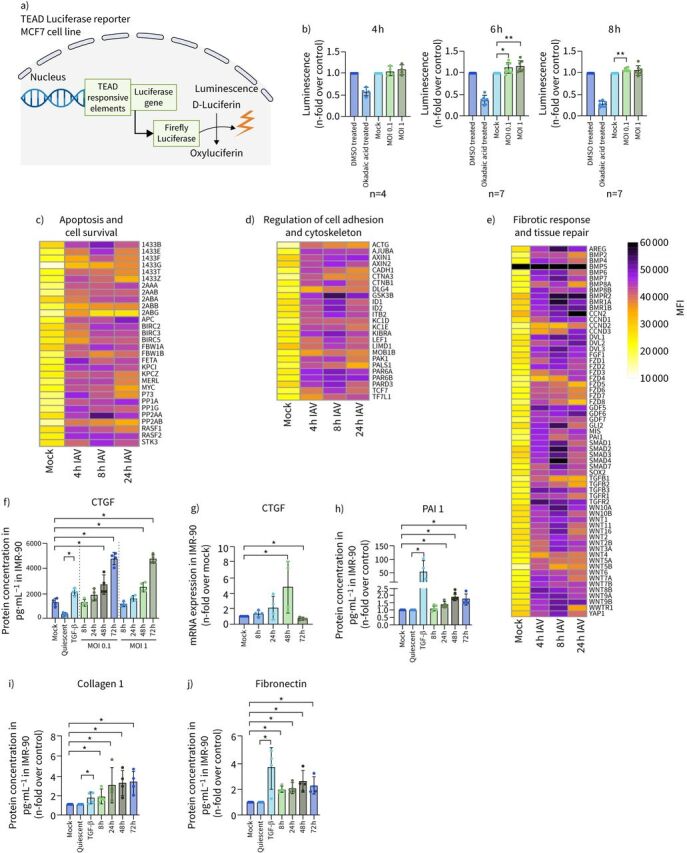
Influenza A virus (IAV) infection in lung fibroblast leads to higher levels of Collagen 1, Fibronectin, PAI1 and CTGF indicating a fibrotic phenotype. a) Schematic illustration of TEAD Luciferase reporter cell line. b) TEAD Luciferase reporter cell line was infected with IAV with multiplicity of infection (MOI) 0.1 and MOI 1 for 4, 6 and 8 h. Luciferase activity was measured by luminescence and normalised to the respective control (n=4 (4 h), n=7 (6 h, 8 h)). c–e) Hippo Pathway Screening Array Assay of IMR-90 cells infected with IAV MOI 0.1 was performed. Heatmaps show protein levels of Hippo signalling pathway proteins concerning apoptosis and cell survival, regulation of adhesion and cytoskeleton as well as fibrotic development (n=4). Median fluorescence intensity (MFI) describes degree of change using purple for higher and yellow for lower protein levels. f) Protein levels of CTGF in mock and infected IMR-90 cells (MOI 0.1 and MOI 1) were measured by ELISA (n=4). g) Expression of CTGF in mock and infected IMR-90 cells (MOI 0.1) was analysed using qPCR, and the fold change compared to the respective control is shown (n=4). h) Protein levels of PAI-1 in mock- and infected IMR-90 cells (MOI 0.1) were measured by ELISA, and the fold change compared to the respective control is shown (n=4). i–j) Protein levels of Collagen 1 and Fibronectin in mock and infected IMR-90 cells (MOI 0.1) were quantified using Western blotting. The fold change compared to the respective control is shown (n=4). Data are expressed as mean±sd, and statistical significance was calculated with the Mann–Whitney U-test (f–j) and Kruskal–Wallis test (b). TEAD: TEA domain family member; CTGF: connective tissue growth factor; PAI-1: plasminogen activator inhibitor-1; TGF-β: transforming growth factor-β. *: p≤0.05; **: p≤0.01.

To assess the interaction of IAV with the Hippo signalling pathway, we used the TEAD luciferase reporter cell line MCF7, in which luciferase expression reflects TEAD activity (figure 5a). Infection with IAV (MOI 0.1 and 1) increased luciferase activity at 6–8 hpi, confirming TEAD activation (figure 5b), consistent with observations in lung fibroblasts.

Proteomic analysis of IAV-infected IMR-90 cells revealed upregulation of Hippo signalling-related proteins, including those involved in apoptosis, cell survival, adhesion and cytoskeletal organisation (figure 5c–d). Importantly, pro-fibrotic effectors such as CTGF and SMAD proteins were elevated (figure 5e). CTGF protein and expression levels, as well as PAI-1, were significantly increased upon infection (figure 5f–h). Furthermore, Collagen 1 and Fibronectin levels were higher in infected cells, indicating a fibrotic phenotype (figure 5i–j, supplementary figure S4B–C).

To determine whether the observed changes in fibrotic markers were mediated through Hippo signalling, IMR-90 cells were treated with the TEAD inhibitor K-975 or the corresponding DMSO control for 48 h. K-975 treatment did not induce additional cytotoxicity compared to DMSO and resulted in reduced TEAD protein levels (supplementary figure S5A–B). Following the 48 h treatment period with the inhibitor, cells were infected with IAV (MOI 0.1) in the presence of the inhibitor (supplementary figure S5C). TEAD inhibition led to a reduction in CTGF, Collagen 1 and Fibronectin protein levels compared to the respective DMSO control (supplementary figure S5D–F).

Overall, these findings demonstrate that IAV infection activates Hippo signalling and promotes fibrotic changes in IMR-90 cells.

## Discussion

Pulmonary fibrosis is increasingly recognised as a major age-associated lung disease, characterised by the progressive accumulation of myofibroblasts and excessive extracellular matrix deposition, ultimately leading to respiratory failure [34]. Despite advances in understanding its pathophysiology, the mechanisms driving fibrotic progression remain incompletely understood [15, 35]. Our data suggest that AMs play a central role in both the initiation and amplification of fibrotic responses. Patient-derived AMs exhibited elevated basal and IAV-induced secretion of pro-fibrotic mediators, including MCP-1 and CCL18, which are known to drive fibroblast activation and extracellular matrix remodelling. Our data also indicated increased collagen expression in AMs from fibrotic patients, consistent with previous reports, though further validation is needed [36]. Additionally, the *FTH1* pseudogene *FTH1P2* was among the top DEGs in both uninfected and infected AMs, suggesting possible alterations in iron metabolism, a pathway linked to fibrosis [37]. These observations indicate that AMs from fibrotic lungs are predisposed to a pro-fibrotic state, a phenotype that is further exacerbated under viral challenge (figure 1).

A limitation of our study is the limited number and heterogeneity of the patient cohort used to generate patient-derived AMs. To overcome this, we adapted a state-of-the-art AML model that recapitulates key phenotypic and transcriptional features of primary AMs [30, 33]. AML cells mounted a robust antiviral response upon IAV infection, and their conditioned media appeared to induce modest fibrotic changes in human lung fibroblasts, including increased expression of fibronectin and CTGF (figures 2 and 3). These results indicate a potential contribution of viral infection to the paracrine effects of macrophage-derived mediators on fibroblast activation, although further experiments are needed to clarify this role.

Interestingly, direct infection of lung fibroblasts further revealed a mechanistic link between IAV and fibrotic remodelling. IAV induced transient inactivation of the Hippo signalling pathway, accompanied by TEAD1 activation and persistent upregulation of pro-fibrotic effectors such as PAI-1 and CTGF (figures 4 and 5). This indicates that, beyond paracrine macrophage signalling, IAV can directly reprogramme fibroblast transcriptional networks to promote a pro-fibrotic phenotype. Notably, Hippo pathway dysregulation was temporally confined, yet the downstream fibrotic response persisted, highlighting a potential “hit-and-run” mechanism in which acute viral infection triggers long-lasting fibrotic changes.

Our findings integrate these observations into a dual-mechanism model: IAV infection exacerbates fibrosis indirectly through macrophage-mediated inflammatory signalling and directly by modulating fibroblast-intrinsic Hippo pathway activity. These results extend previous studies demonstrating the importance of Hippo signalling in alveolar epithelial regeneration and cell competition during fibrosis [38–40], suggesting that viral-induced perturbation of this pathway contributes to the impaired tissue homeostasis observed in fibrotic lungs.

Collectively, our findings underscore the dual role of respiratory viral infections in both exacerbating pre-existing interstitial lung disease and initiating fibrotic remodelling. By delineating macrophage-derived mediators and fibroblast Hippo signalling as key drivers of this process, we identify potential therapeutic entry points. Targeting these pathways may offer strategies to limit virus-induced fibrotic progression.

## Data Availability

The data that support the findings of this study are available from the corresponding author upon reasonable request.

## Supplementary material

**Please note:** supplementary material is not edited by the Editorial Office, and is uploaded as it has been supplied by the author. Supplementary material 01123-2025.supplement Supplementary figures 01123-2025.supplement2 Supplementary tables 01123-2025.supplement
