# “Hippo pathway activation drives fibrogenic remodelling in influenza A virus-infected lung fibroblasts” Yasmina Reisser, Elaine Winkler, Julia Hoffmann, Nilima Dinesh Kumar, Antje Häder, Susanne M. Lang, Bettina Löffler and Stefanie Deinhardt-Emmer. *ERJ Open Res* 2026; 12: 01123-2025.

**DOI:** 10.1183/23120541.51123-2025

**Published:** 2026-07-20

This article was originally published with an error in figure 1b in which one of the data points was inadvertently omitted during the journal's production process. The corrected figure is shown below. This has been corrected in the article itself. We apologise for this error.

**FIGURE 1:**
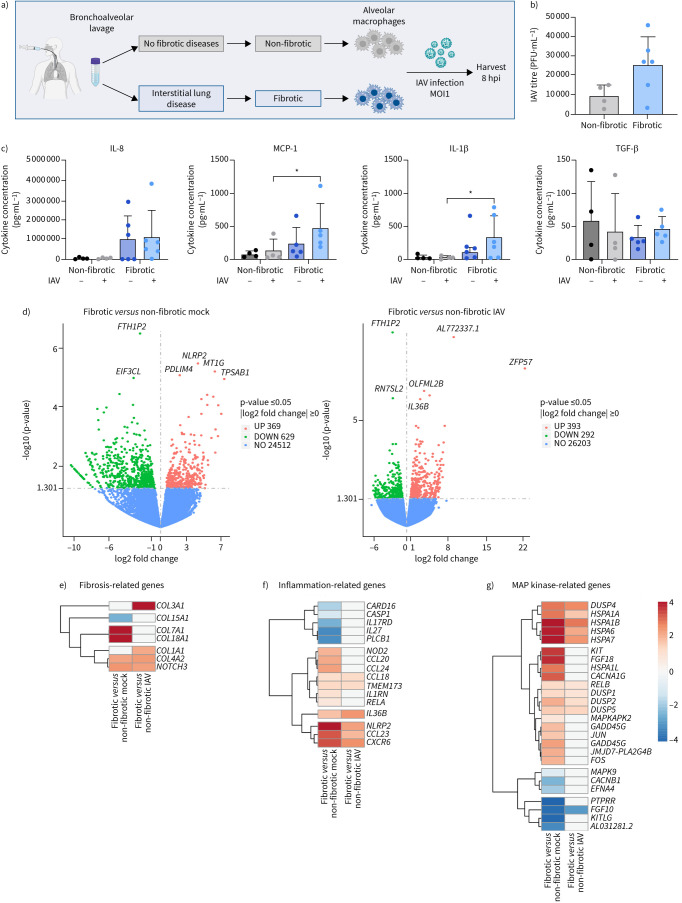
Influenza A virus (IAV) infection of alveolar macrophage (AM) from fibrotic patients results in elevated viral load and inflammatory response. a) Schematic overview of the study design. Patients who received a bronchoalveolar lavage (BAL) were grouped in fibrotic (n=6) and nonfibrotic (n=4) groups based on clinical data. AM were subsequently isolated from the BAL and infected with IAV with a multiplicity of infection (MOI) of 1 for 8 h. b) Viral titre in the supernatant of AM from fibrotic (n=6) and nonfibrotic (n=4) patients at 8 hpi, determined by plaque assay. IAV titers were normalised to cell count and expressed as PFU·mL^−1^, adjusted to 200 000 cells. c) Cytokine levels in the supernatant of mock- and IAV-infected AMs from fibrotic (n=6; unless outliers were removed) and nonfibrotic patients (n=4; unless outliers were removed) were measured. Values were normalised to cell count and expressed as pg·mL^−1^, adjusted to 200 000 cells. d) Volcano plots displaying differentially expressed genes (DEGs), with upregulated genes shown in red and downregulated genes in green. For each comparison, the six most significant DEGs are highlighted. Significance was defined as p≤0.05. Three biological replicates of patient-derived AMs were sequenced per group. e–g) Heatmaps depicting the log_2_ fold changes of DEGs comparing fibrotic and nonfibrotic samples under mock- and IAV-infected conditions. Genes associated with fibrosis (e), inflammation (f) and the MAP kinase pathway (g) are shown. Upregulated genes are displayed as red, while downregulated genes are displayed as blue. Log_2_ fold change of genes, which were not differentially expressed, was set to 0 and are displayed as white. Each data point represents an individual biological replicate. Data are shown as mean±sd. Statistical significance was assessed using the Mann–Whitney U-test (b) and Kruskal–Wallis test (c) (*p≤0.05). For c, outliers were identified and removed using the ROUT method. PFU: plaque-forming unit; hpi: hours post-infection; IL: interleukin; TGF-β: transforming growth factor-β.

