# Supplementary material for: Hippo pathway activation drives fibrogenic remodelling in influenza A virus-infected lung fibroblasts
Source: ERJ Open Res. 2026 Jun 17;12(3):01123-2025. doi: 10.1183/23120541.01123-2025 (PMC13266427; doi:10.1183/23120541.01123-2025)
Supplement: Supplementary file 1 [file 01123-2025.supplement.pdf]

Supplementary Materials and Methods

Isolation of patient derived alveolar macrophages from BAL

In short, BAL fluid was centrifuged at 250 x g for 10 min at 4 °C, and the cell pellet was washed with Dulbecco's phosphate-buffered saline (DPBS, Thermo Fisher Scientific, Waltham, Massachusetts, USA) supplemented with 2% FBS and 2mM ethylenediaminetetraacetate (EDTA, Sigma-Aldrich, Taufkirchen, Germany). The cell pellet was resuspended in culture medium consisting of Roswell Park Memorial Institute 1640 (RPMI, Thermo Fisher Scientific, Waltham, Massachusetts, USA) containing 10% FBS, 1% P/S, 1 mM sodium pyruvate (Thermo Fisher Scientific, Waltham, Massachusetts, USA) and 10 mM N-2-hydroxyethylpiperazine-N'-2-ethanesulphonic acid (HEPES; Thermo Fisher Scientific, Waltham, Massachusetts, USA). Subsequently, cells were counted, seeded and incubated for 2 h at 37°C and 5% CO_2, before changing medium to fresh culture medium. Cells were used for infection experiment on the following day.

Fibroblast-to-myofibroblast transformation assay

For fibroblast-to-myofibroblast transformation assay, IMR-90 were seeded in DMEM with 10% FBS, 1%P/S. Media was changed to DMEM with 0.2% FBS on day 1 and to serum-free DMEM on day 2. On day 3 cells were treated with 1.25 ng/mL TGF-β1 (Sigma Aldrich, Taufkirchen, Germany) in DMEM. Quiescent cells were generated by serum starvation as previously published and used as control cells [34]. Quiescent cells were harvested on day 4 and TGF- β1 treated cells on day 6.

Isolation of peripheral blood mononuclear cells

For isolation of peripheral blood mononuclear cells (PBMCs), buffy coats were diluted 1:4 in DPBS, 2 mM EDTA, and 0.1% bovine serum albumin (BSA, Carl Roth GmbH) and layered onto

Histopaque-1077 (Sigma Aldrich). After centrifugation at 800 × g for 20 min at room temperature (RT), the PBMC layer was collected and washed three times with DPBS, 2 mM EDTA, and 0.1% BSA. Monocytes were purified from PBMCs using $CD14^+$ magnetic bead selection (Miltenyi Biotec) following the manufacturer's instructions.

Differentiation into monocyte-derived macrophage control and alveolar macrophage-like cells

For alveolar macrophage-like (AML) cells differentiation was performed as previously described [35]. Cells were cultured in RPMI 1640 medium supplemented with 10% human serum, 1% P/S, and a differentiation cocktail comprising 10 ng/mL GM-CSF, 5 ng/mL transforming growth factor-beta (TGF-β, Biolegend, San Diego, California, USA), 5 ng/mL interleukin-10 (IL-10, BD Biosciences, Franklin Lakes, New Jersey, USA), and 100µg/mL Curosurf (poractant alfa, Chiesi, Parma, Italy).

For both MDM and AML, cells were cultured at a density of 2×10^6 cells/mL in 6-well suspension plates (Greiner Bio-One, Kremsmünster, Austria), pre-treated with Anti-Adherence Rinsing Solution (STEMCELL Technologies, Vancouver, Canada) at 37°C in a humidified atmosphere with 5% CO_2 for six days, with GM-CSF or the complete differentiation cocktail replenished every three days for MDM and AML cells respectively.

Infection and conditioned media experiments

Patient-derived AM were infected with IAV at a multiplicity of infection (MOI) of 1. IMR-90 were infected at MOIs of 0.1 and 1, as indicated. Cells were incubated for 30 min at 37°C and 5% CO_2 with viral dilutions in DPBS containing 0.2% BSA, 1 mM $MgCl_2$ and 0.9 mM $CaCl_2$ (both Sigma Aldrich, Taufkirchen, Germany). After washing with DPBS, AM were incubated in RPMI (0.2% BSA, 1 mM $MgCl_2$, 0.9 mM $CaCl_2$, 30 ng *L*-1-tosylamido-2-phenylethyl

chloromethyl ketone (TPCK)-treated trypsin (Thermo Fisher Scientific, Waltham, Massachusetts, USA)) for 8 h. IMR-90 were treated similarly but incubated in DMEM (0.2% BSA, 1 mM $MgCl_2$, 0.9 mM $CaCl_2$ and 30 ng TPCK-treated trypsin) for 8, 24, 48, and 72 h.

Hippo Pathway TEAD Reporter - MCF7 cells were infected with IAV at an MOI of 0.1 and 1. Cells were incubated for 30 min at 37°C and 5% CO_2 with viral dilutions in EMEM supplemented with 10% FBS, 1% NEAA, 1 mM sodium pyruvate, 1% P/S, 10 µg/mL insulin, 0.2% BSA, 1 mM $MgCl_2$ and 0.9 mM $CaCl_2$. After washing with DPBS, cells were incubated in EMEM supplemented with 10% FBS, 1% NEAA, 1 mM sodium pyruvate, 1% P/S, 10 µg/mL insulin, 0.2% BSA, 1 mM $MgCl_2$, 0.9 mM $CaCl_2$ and 30 ng TPCK-treated trypsin for 4 h, 6 h and 8 h.

Fully differentiated AML cells were maintained for two additional days in tissue culture dishes, and the supernatants were collected for use as conditioned media (CM). To control for potential effects of the media and cytokines, AML media without cells was used as a control. IMR-90 were treated with DMEM (10% FCS, 1% P/S), AML media, or CM from AML cells (diluted 1:1 in DMEM with 10% FCS, 1% P/S) for 8, 24 and 48h.

After infection of AML cells with IAV at an MOI of 1, supernatants were collected at 8 and 24 h, UV-inactivated for 30 min, and used to treat IMR-90 as described above.

RNA Extraction, cDNA Synthesis, and qRT-PCR

Cultured cells were washed and lysed in RLT buffer (Qiagen, Hilden, Germany). Isolating of total RNA was performed by using the Qiagen RNeasy Mini Kit (Qiagen, Hilden, Germany) according to the manufacturer's protocol. RNA concentration and purity was determined using a NanoDrop spectrophotometer ND-1000 (Peqlab/VWR, Radnor, USA). cDNA synthesis was performed using the High-Capacity cDNA Reverse Transcription Kit (Thermo Fisher

Scientific, Waltham, Massachusetts, USA). For qRT-PCR (95 °C for 10 min, followed by 45 cycles of 95°C for 10 s, 60°C for 20 s, and 72°C for 30 s) a Maxima SYBR Green qPCR Master Mix (Thermo Fisher Scientific) and Rotor-Gene Q (Qiagen, Hilden, Germany) were used. Primers were all obtained from metabion (Planegg, Germany). *ACTB* and *GAPDH* were used as housekeeping genes. Expression of target genes is shown as n-fold change of the appropriate control.

Viral RNA was extracted from cell culture supernatants and cell lysates of AML cells using the QIAamp viral RNA mini kit (Qiagen, Hilden, Germany). The qRT-PCR for IAV was performed using the RIDA GENE flu kit (R-Biopharm AG, Darmstadt, Germany) according to the manufacturer's instructions and measured in a Rotor-Gene Q (Qiagen, Hilden, Germany).

mRNA sequencing

RNA concentration and purity of all samples was determined using a NanoDrop spectrophotometer ND-1000 prior to sequencing.

Following quality control and sequencing, the bioinformatic analysis included the following steps: Raw FASTQ reads were processed with fastp to remove adapters, poly-N sequences, and low-quality reads. Raw FASTQ reads were processed with fastp to remove adapters, poly-N sequences, and low-quality reads. Clean reads were mapped to the *Homo sapiens* reference genome (GRCh38/hg38) using HISAT2. Gene expression was quantified using the fragments per kilobase of transcript per million fragments mapped (FPKM) method. Differential expression analysis was performed with DESeq2, and false discovery rate (FDR) correction was applied using the Benjamini-Hochberg procedure.

Heatmap construction for visualization of DEGs was conducted using R studio (version 4.3.1, R Foundation for Statistical Computing, https://www.r-project.org/). The log2fold change of

each depicted gene is displayed for the indicated comparison. All genes displayed have a pvalue ≤0.05 for at least one of the comparisons. The log2fold change of genes which were not significant for a comparison was set to 0 and depicted as white in the heatmaps.

Protein extraction and Western blot

Cells were lysed in RIPA buffer containing Halt Protease and Phosphatase Inhibitor Cocktail (Thermo Fisher Scientific, Waltham, Massachusetts, USA), vortexed every 10 min for 3 times, centrifuged at 10.000 x g at 4°C for 10 min and supernatants were collected. Micro BCA Protein Assay Kit (Thermo Fisher Scientific, Waltham, Massachusetts, USA) was used to determine protein concentrations. Afterwards extracted proteins were run on SDS-PAGE and blotted onto polyvinylidene fluoride membranes (PVDF, Thermo Fisher Scientific, Waltham, Massachusetts, USA). The following primary antibodies were used: Large tumor suppressor kinase 1 (LATS 1; 1:500), phosphorylated LATS 1 (pLATS 1; 1:500), YAP/TAZ (1:1000), phosphorylated YAP/TAZ (pYAP/pTAZ; 1:1000), TEA domain family member 1 (TEAD 1 ; 1:1000), and Fibronectin (1:1000 for IMR-90, 1:5000 for IMR-90 with CM) (all from Cell Signalling Technology, Danvers, Massachusetts, USA); Collagen 1 (1:1000 for IMR-90, 1:15000 for IMR-90 with CM) (Abcam, Cambridge, UK)). Membranes were incubated with horseradish peroxidase (HRP)-conjugated anti-rabbit IgG (Bio-Rad, Hercules, California, USA) HRP-β-Actin, and HRP-Vinculin (both santa cruz biotechnology, Dallas, Texas, USA) as housekeeping proteins. All secondary antibodies were used at 1:1000. Visualisation and quantification was performed using Immobilon Western HRP substrate (Merck Millipore, Darmstadt, Germany) on a iBright™ CL750 Imageing System (Thermo Fisher Scientific, Waltham, Massachusetts, USA).

Immunofluorescence

AML cells were cultured in 24-well plates, fixed with 4% PFA (Sigma Aldrich, Taufkirchen, Germany) for 15 min at 37°C, permeabilised with 0.1 % Triton-X (Roth, Karlsruhe, Germany) in DPBS and blocked with 3% BSA in DPBS for 30 min. Plates were incubated with IAV nucleoprotein (1:1000, Bio-Rad, Hercules, California, USA) diluted in blocking solution at RT for 1h. Alexa Fluor® 488 AffiniPure Goat Anti-Mouse IgG (H+L) (Jackson Immuno Research, Cambridge, UK, 1:500) and Alexa Fluor® Plus 488 or 647 Phalloidin (1:400, Thermo Fisher Scientific, Waltham, Massachusetts, USA) were used as secondary antibody. Visualisation was carried out using the All-in-One Fluorescence Microscope BZ-X810 (Keyence, Osaka, Japan).

Flow Cytometry

MDM and AML cells were detached using cell dissociation buffer (Thermo Fisher Scientific, Waltham, Massachusetts, USA) and scraping. The cells were then transferred to FACS tubes, centrifuged at 250 × g for 5 min at 4°C, and resuspended in DPBS. Viability was assessed by staining with 1:1000 diluted BD Fixable Viability Stain 780 (BD Biosciences, Heidelberg, Germany) for 15 min at RT. To block Fc receptors, cells were incubated with human TruStain FcX™ (1:50, BioLegend). Surface marker staining was performed using the following antibodies: anti-MARCO (1:20, Thermo Fisher Scientific, Waltham, Massachusetts, USA), anti-CD36 and anti-CD11b (both 1:50, BioLegend, San Diego, California, USA) (Supplementary Table 2).

Hippo Pathway Screening Array Assay

IMR-90 cells were washed with DPBS and lysed in RIPA buffer containing Halt Protease and Phosphatase Inhibitor Cocktail. The quantification of 118 proteins from the Hippo signalling pathway was performed by Creative Proteomics (Shirley, New York, USA) via protein assay. The Hippo Pathway Screening Array Assay is a semi-quantitative screen for the relative levels

of human proteins involved in the Hippo signalling pathway. In this assay, proteins in the cell lysates were biotinylated to label primary amine groups. The biotin-labeled sample was incubated on a pre-blocked glass slide array containing capture antibodies, allowing target proteins to bind. Streptavidin-conjugated Cy3 fluorescent dye was applied, and after drying, laser fluorescence scanning was used to visualize signals. Each array includes three Positive Controls (standardized biotinylated IgGs), which enable normalization by providing consistent fluorescence signals across arrays. Images were captured with an Axon Innopsys InnoScan 710 AL laser scanner. The results are median fluorescent signals at the 532 wavelengths.
