# Supplementary material for: Hippo pathway activation drives fibrogenic remodelling in influenza A virus-infected lung fibroblasts
Source: ERJ Open Res. 2026 Jun 17;12(3):01123-2025. doi: 10.1183/23120541.01123-2025 (PMC13266427; doi:10.1183/23120541.01123-2025)
Supplement: Supplementary file 2 [file 01123-2025.supplement2.pdf]

Supplementary Figure 1

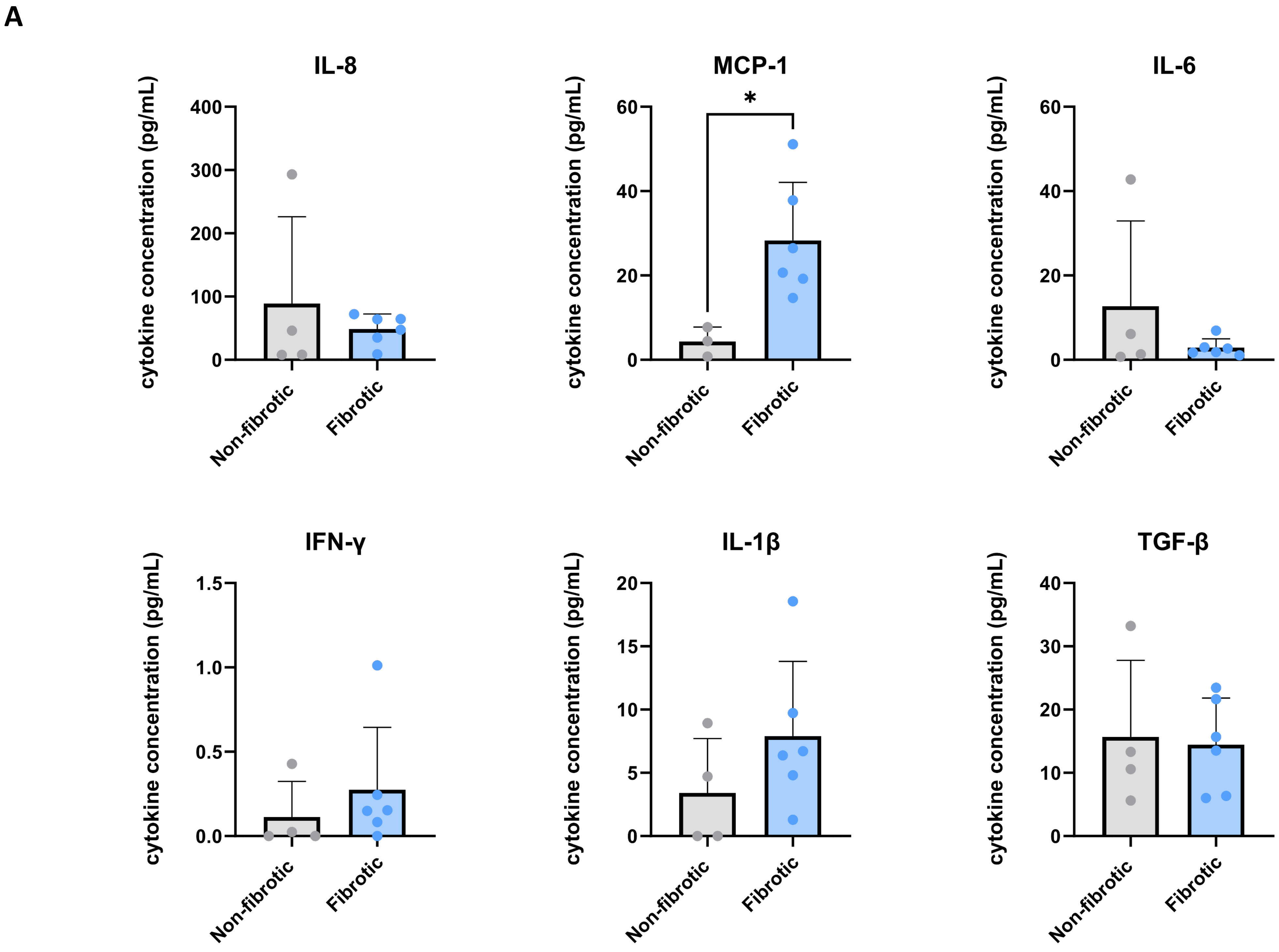

Supplementary Figure 2

A

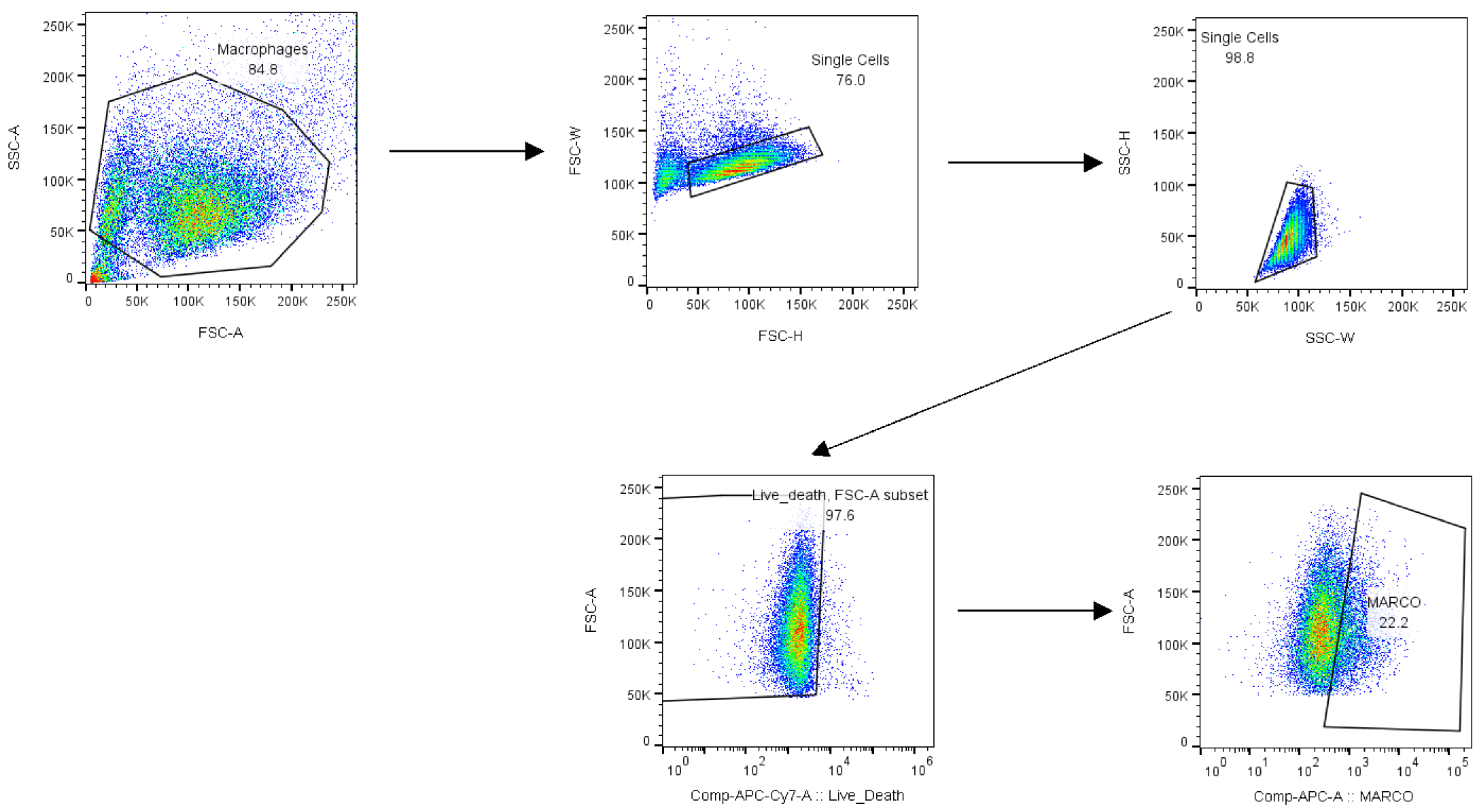

B

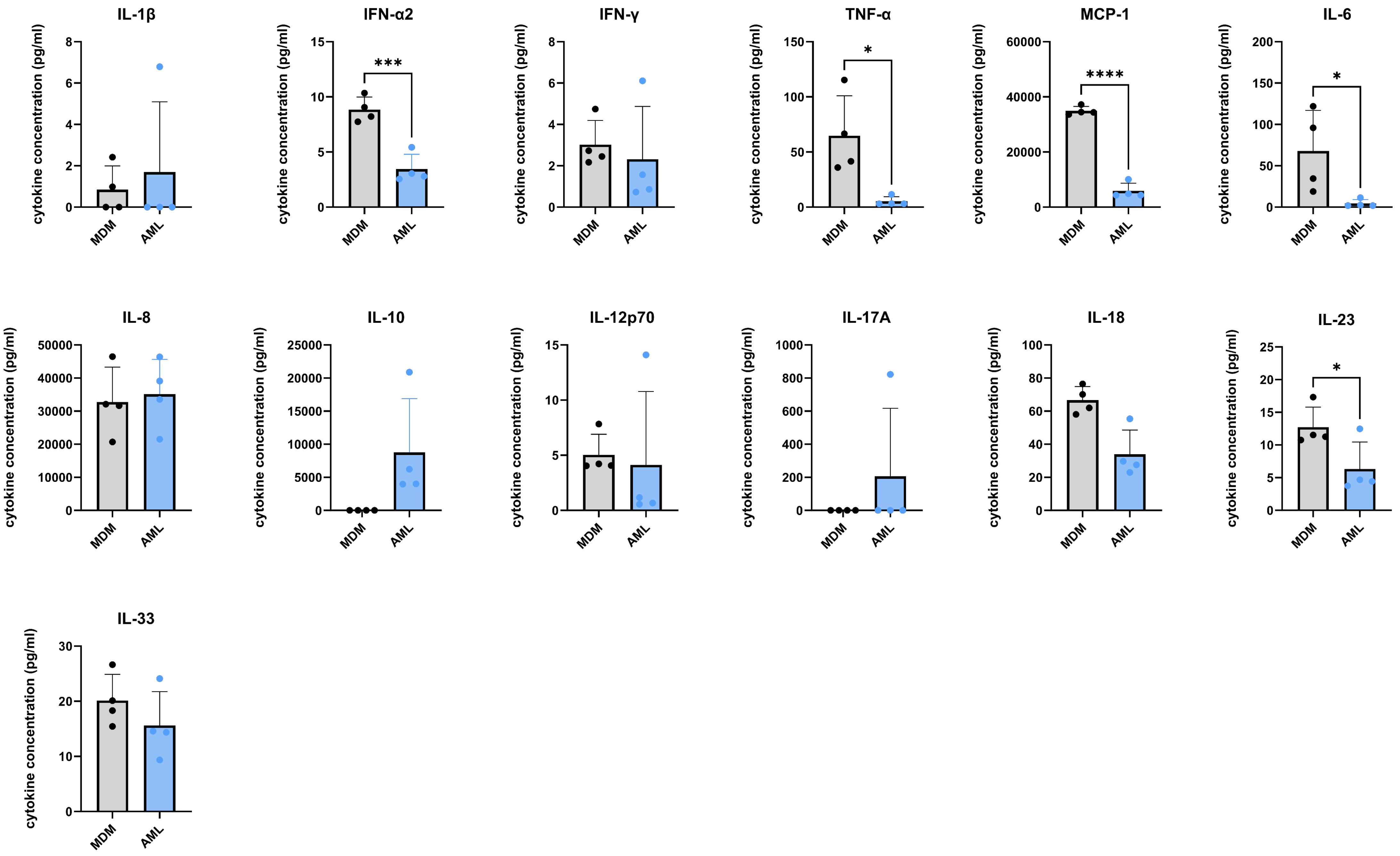

Supplementary Figure 2

C

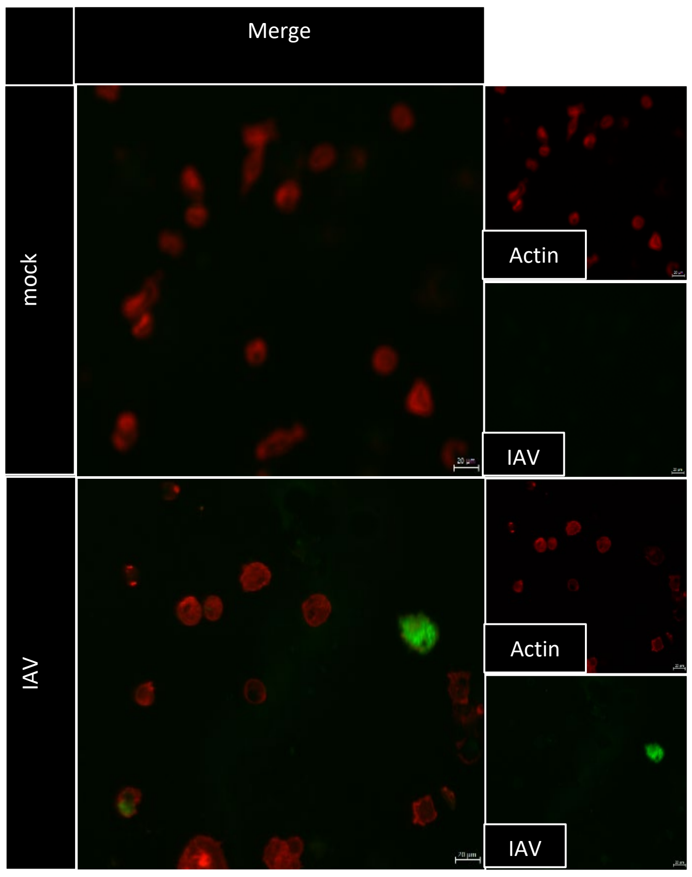

D

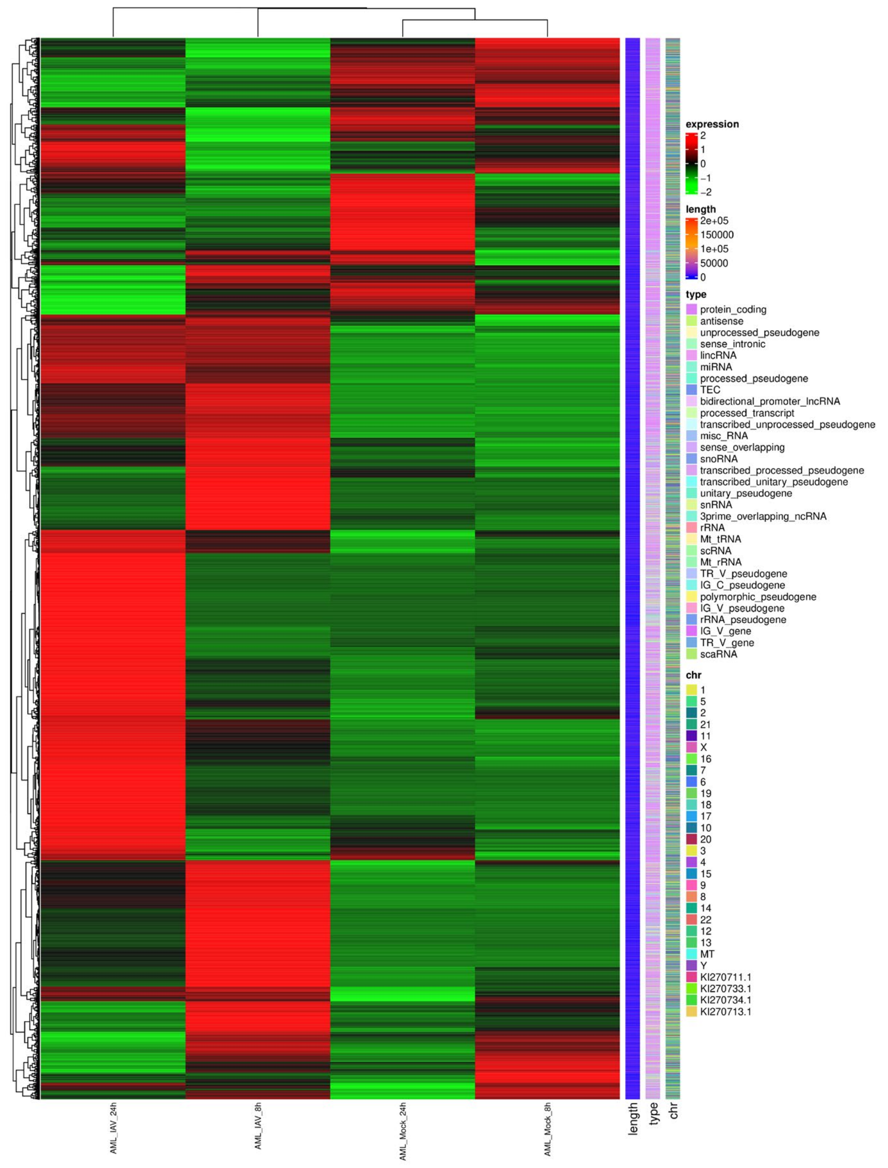

E

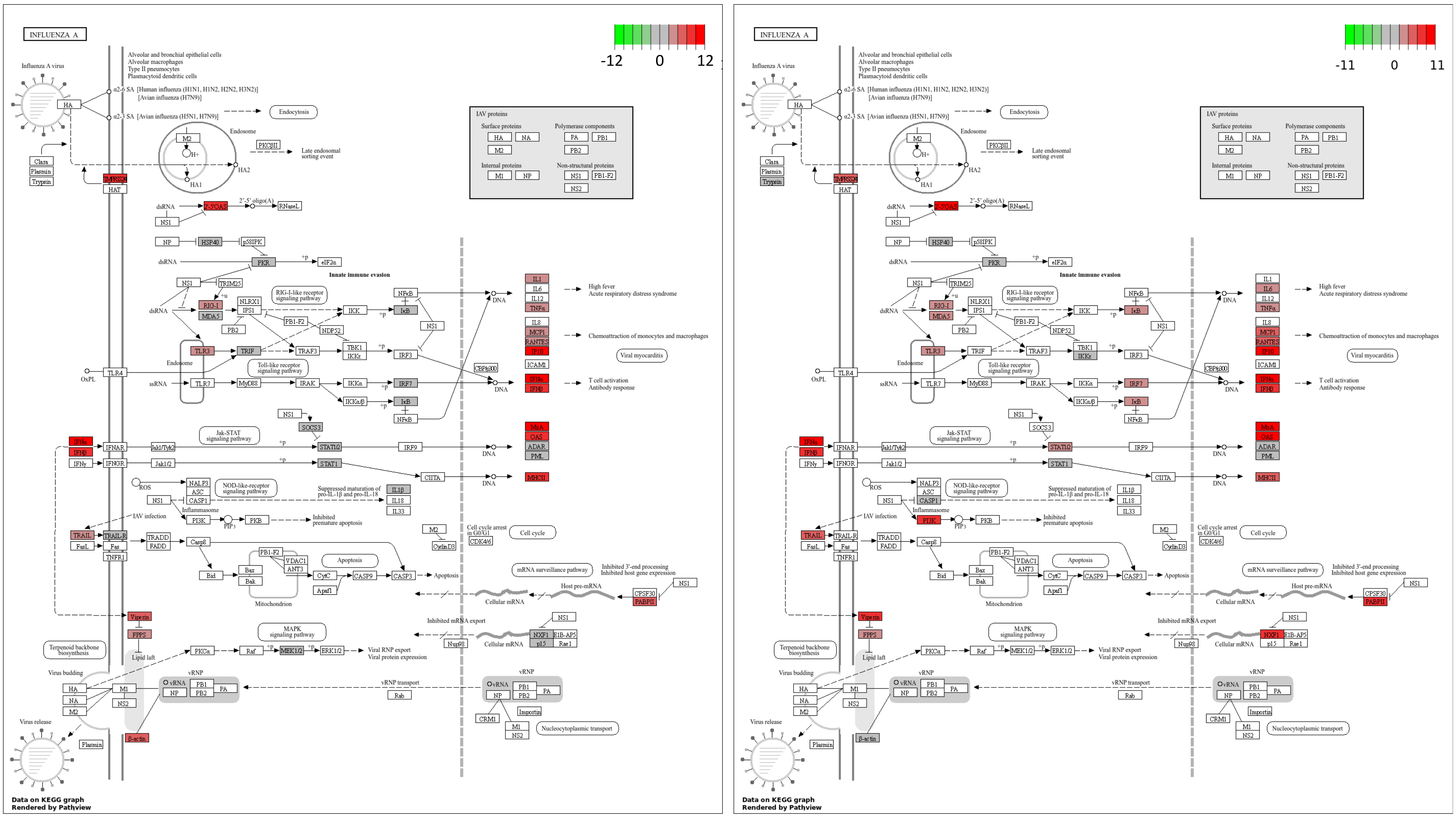

F

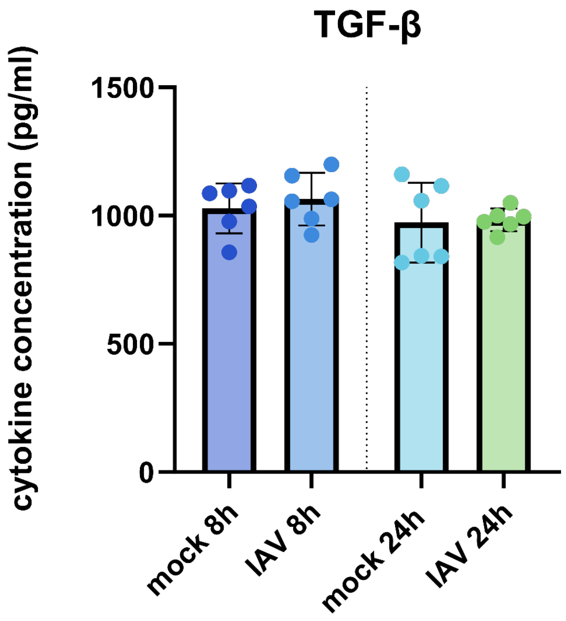

Supplementary Figure 3

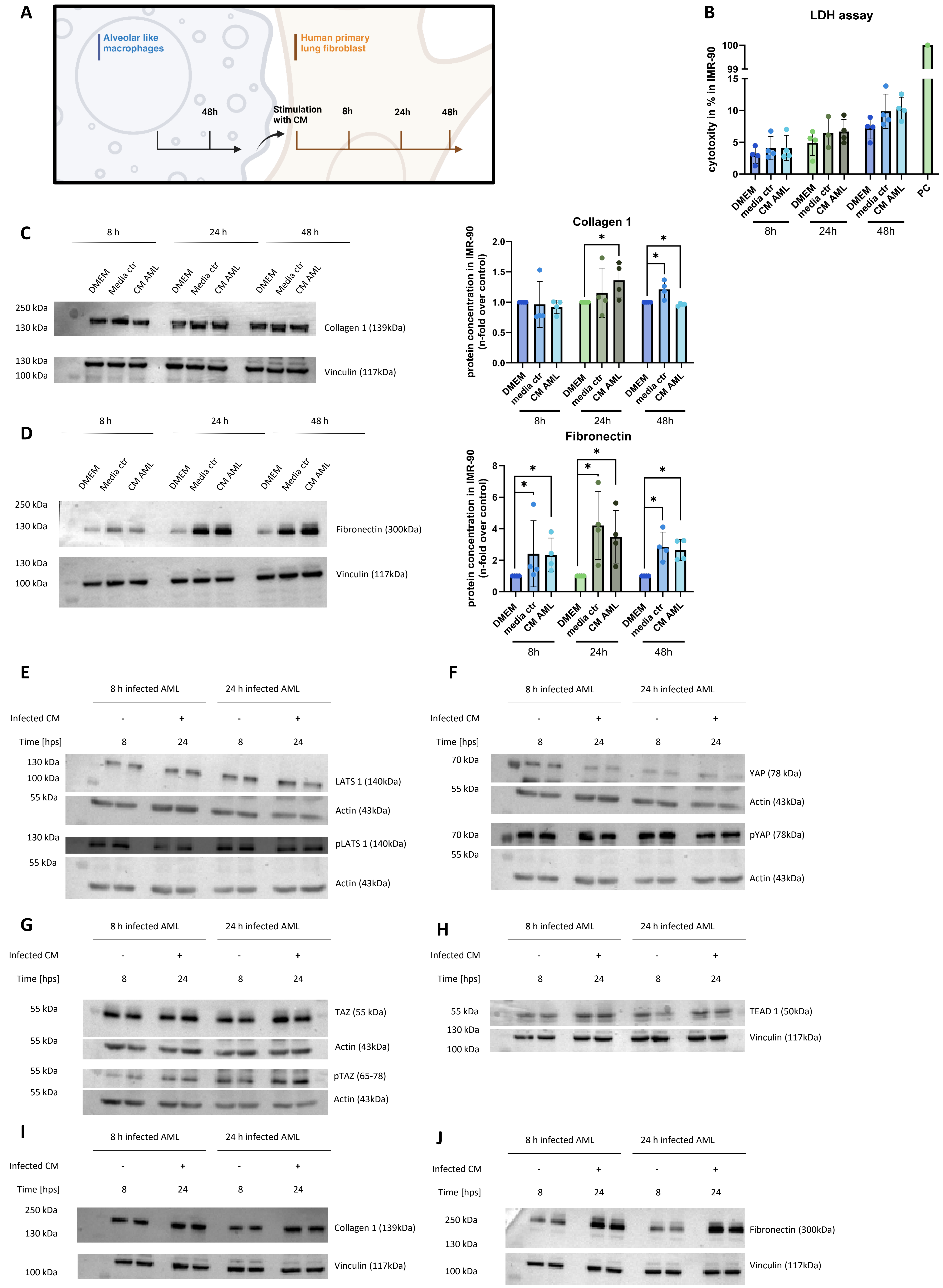

Supplementary Figure 4

A

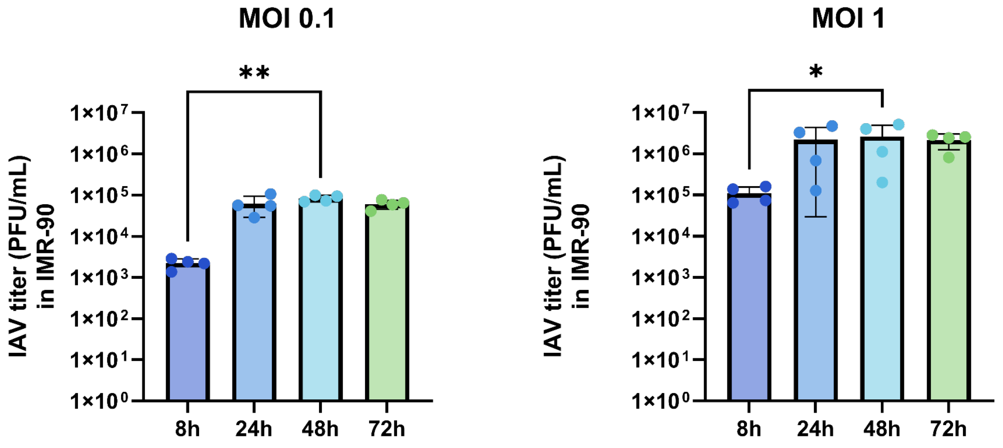

B

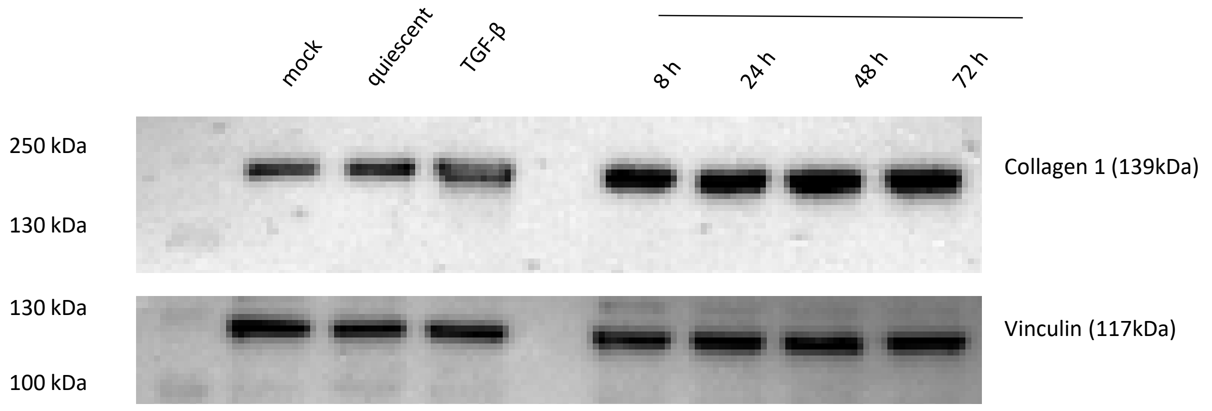

C

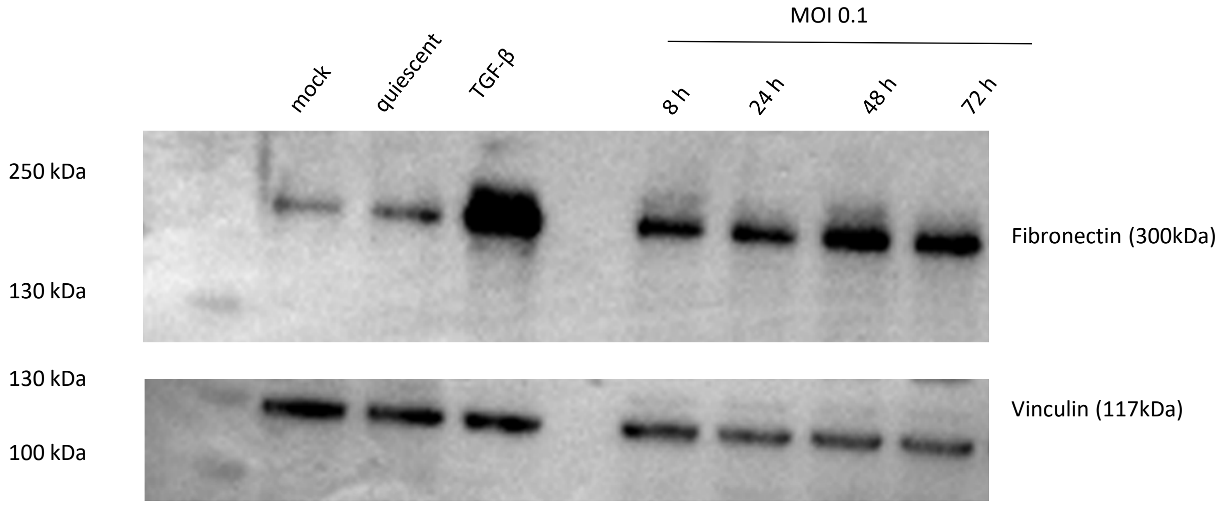

Supplementary Figure 5

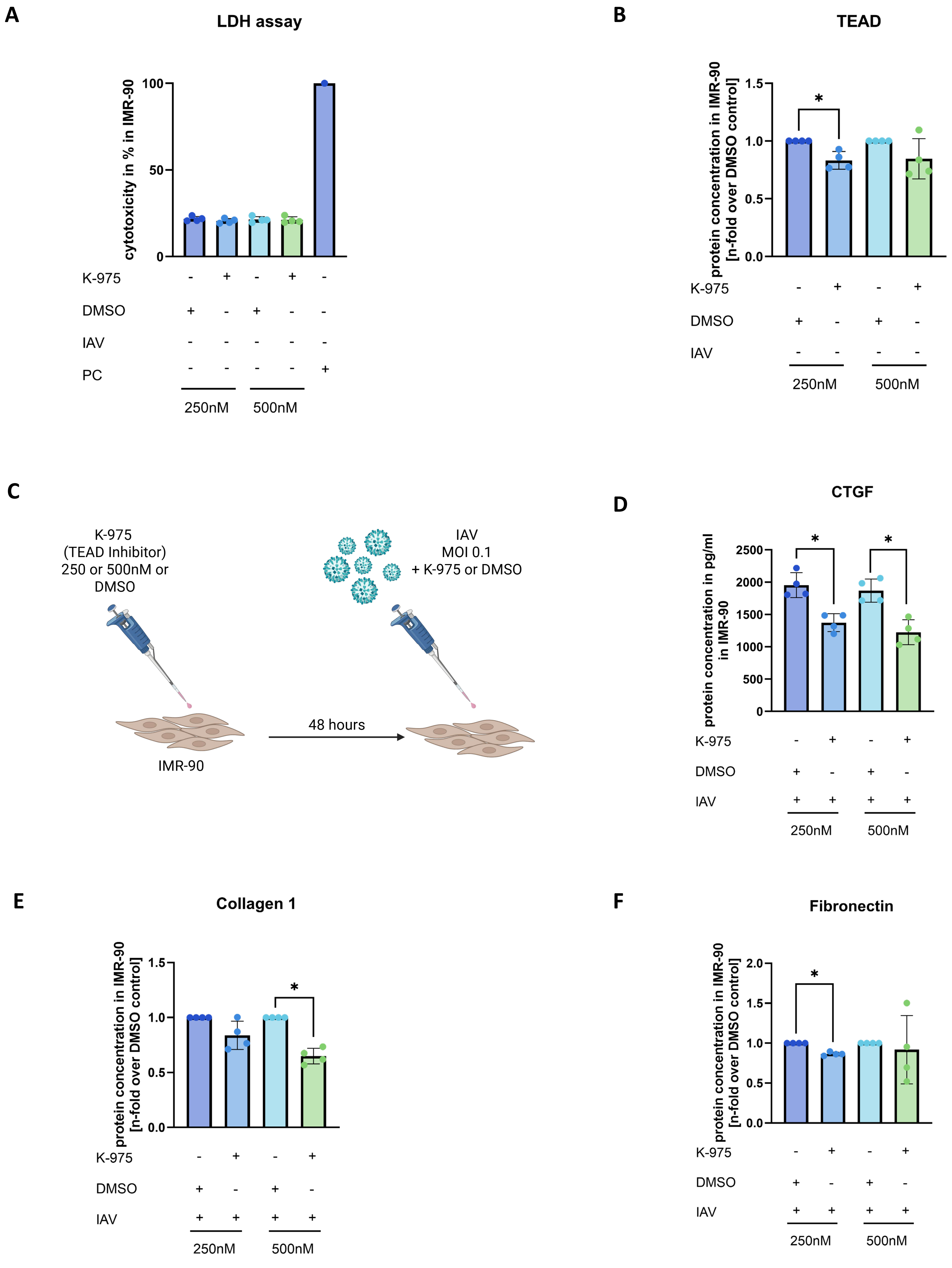

Figure Legends

Supplementary Figure 1.

(A) Cytokine levels in the BAL of fibrotic (n = 6) and non-fibrotic (n = 4) patients, except for MCP-1 where 1 outlier for the non-fibrotic group was identified and removed. Cytokine levels were normalised to BAL cell count and expressed as pg/mL, adjusted to 1 million cells.

Each data point represents an individual biological replicate. Data are shown as mean ± SD. Significance was calculated with the Mann-Whitney U test (*p ≤ 0.05). Outliers were identified and removed using the ROUT method. BAL = Bronchoalveolar lavage

Supplementary Figure 2

(A) Gating strategy for flow cytometry analysis. Cells were first gated on FSC/SSC to exclude debris. Single cells were then identified by gating FSC-height vs. FSC-width and SSC-width vs. SSC-height to eliminate doublets. Viable cells were defined as negative for the viability dye, and MARCO-positive cells were gated from live, single-cell populations. (B) Cytokine levels in the MDM and AML cells measured in the supernatants (n = 4). (C) Immunofluorescence staining of infected AML cells (24 hpi) with antibody against IAV NP (green), phalloidin (red). Scale bar: 20 µM. (D) Heatmap of overall gene expression in AML cells at 8 and 24 hpi compared to the mock treatment (n = 6). Red indicates upregulation, and green indicates downregulation of the gene. (E) KEGG- pathway enrichment analysis of the influenza pathway (n = 6) comparing mock treatment to IAV-infected for 8 and 24 hpi. Red signals upregulation, green signal downregulation. (F) TGF-β levels in IAV-infected AML cells were measured using multiplex cytokine assay (n = 6). Data are expressed as mean ± SD, and statistical significance was determined using an unpaired Student's t-test (A) and Mann-Whitney U test (F) (*P ≤ 0.05, **P ≤ 0.01, ***P ≤ 0.001, ****P ≤ 0.0001. MDM = Monocyte-Derived Macrophages, AML = alveolar macrophage-like, FSC=Forward Scatter, SSC = Side Scatter, MARCO = Macrophage Receptor with Collagenous Structure, IAV NP = Influenza A Virus Nucleoprotein, hpi = hours post-infection, KEGG = Kyoto Encyclopedia of Genes and Genomes, TGF-β =Transforming Growth Factor Beta, SD = Standard Deviation

Supplementary Figure 3

(A) Schematic overview of stimulation experiment. Supernatants of AML cells were collected 2 days after differentiation. IMR-90 cells were treated with DMEM or AML cell supernatants for 8, 24 or 48 h. (B) Cytotoxicity of IMR-90 cells treated with DMEM, AML media, or AML cell supernatants (after 2 days of culture) was measured at 8, 24, and 48 h using an LDH assay.

(C-D) Protein levels of Collagen 1 and Fibronectin in IMR-90 cells treated with CM without infection were quantified using Western blotting (n = 4). Protein levels were normalised to the DMEM medium control. A representative blot is shown. (E) Representative Western blot used for quantification of LATS 1 and pLATS 1 protein levels in IMR-90 cells treated with infected CM (n = 4). (F) Representative Western blot used for quantification of YAP and pYAP protein levels in IMR-90 cells treated with infected CM (n = 4). (G) Representative Western blot used for quantification of TAZ and pTAZ protein levels in IMR-90 cells treated with infected CM (n = 4). (H) Representative Western blot used for quantification of TEAD 1 protein levels in IMR-90 cells treated with infected CM (n = 4). (I) Representative Western blot used for quantification of Collagen 1 protein levels in IMR-90 cells treated with infected CM (n = 4). (J) Representative Western blot used for quantification of Fibronectin protein levels in IMR-90 cells treated with infected CM (n = 4). Significance was calculated with the Mann-Whitney U test (B-D) (* p ≤ 0.05). AML = alveolar macrophage-like, CM = conditioned media, LDH = Lactate dehydrogenase, LATS 1 = Large tumor suppressor kinase 1, YAP = Yes-associated protein, TAZ = Transcriptional coactivator with PDZ-binding motif, TEAD = TEA domain family member, CTGF = Connective tissue growth factor

Supplementary Figure 4

(A) Quantification of viral titer of IMR-90 cells infected with IAV at the indicated MOI in PFU/mL using standard plaque assay (n = 4). (B) Representative Western blots for the quantification of (B) Collagen 1 and (C) Fibronectin protein levels in IAV-infected IMR-90 cells (n = 4). Data are expressed as mean ± SD, and statistical significance was calculated using Kruskal-Wallis test (A) (*p ≤ 0.05, **p ≤ 0.01). MOI = Multiplicity of infection, PFU = plaque-forming units, IAV = Influenza A virus

Supplementary Figure 5

(A) Cytotoxicity of IMR-90 cells treated with DMSO or the TEAD inhibitor K-975 for 48 h was assessed using an LDH assay (n = 4). (B) Protein levels of TEAD in uninfected IMR-90 cells treated with K-975 were quantified by Western blotting, and the fold change relative to the respective DMSO control is shown (n = 4). (C) Schematic representation of the TEAD inhibition and infection workflow. IMR-90 cells were treated with DMSO or K-975 for 48 h, followed by infection with IAV (MOI 0.1) for 24 h. The respective treatment (DMSO or K-975) was maintained during infection. (D) Protein levels of CTGF in infected IMR-90 cells (MOI 0.1) treated with DMSO or K-975 were measured by ELISA (n = 4). (E–F) Protein levels of Collagen 1 and Fibronectin in infected IMR-90 cells (MOI 0.1) treated with DMSO or K-975 were quantified using Western blotting. The fold change relative to the respective DMSO control is shown (n = 4). Data are expressed as mean ± SD, and statistical significance was calculated using the Mann–Whitney U test (B, D–F) (*p ≤ 0.05). TEAD = TEA domain family member; IAV = Influenza A virus; MOI = Multiplicity of infection; CTGF = Connective tissue growth factor; DMSO = Dimethyl sulfoxide; LDH = Lactate dehydrogenase; SD = Standard deviation
